# Implications of Timanian thrust systems in the Barents Sea and Svalbard on using paleontological constraints for plate tectonics reconstructions

**DOI:** 10.12688/openreseurope.16674.2

**Published:** 2024-07-12

**Authors:** Jean-Baptiste P. Koehl

**Affiliations:** 1 Earth and Planetary Sciences, McGill University, Montreal, Québec, H3A 0E8, Canada; 2 Geosciences, Universitetet i Oslo, Oslo, Oslo, 0371, Norway

**Keywords:** Svalbard, Laurentia, Baltica, Timanian Orogeny, Caledonian Orogeny, thrust, faunal barrier, trilobite, climate belt, faunal recruitment, Neoproterozoic, Cambrian, Ordovician

## Abstract

**Background:**

The Svalbard Archipelago is commonly believed to have been located at comparable latitude and, possibly, to have been attached to Laurentia in the early Paleozoic (500–420 Ma) based on trilobite assemblage similarities. Trilobite assemblage differences and lack of mixing between Laurentia–Svalbard and Baltica were further used to propose that these continents were separated by the Iapetus Ocean at that time. However, recent structural correlation of Timanian (650–550 Ma) thrust systems throughout the Barents Sea show that Svalbard was already attached to Baltica in the latest Neoproterozoic and remained so during the Phanerozoic.

**Methods:**

The present study presents a new interpretation of seismic reflection data from the DISKOS database, which were tied to nearby exploration wells. The study uses recently acquired knowledge of the seismic facies of intensely deformed pre-Caledonian rocks and principles of seismic stratigraphy to interpret the data.

**Results:**

The present study reconciles the proximity of Svalbard and Laurentia with the early accretion of Svalbard to Baltica in the latest Neoproterozoic. It also describes the influence of Timanian thrust systems on paleoenvironments and possible effects on trilobite assemblages, *e.g.*, the lack of mixing between those of Laurentia–Svalbard and Baltica.

**Conclusions:**

The identification of elongate, emerged topographic highs in the Barents Sea and Svalbard in the late Neoproterozoic–early Paleozoic suggest that paleontological constraints should be considered with greater care when discussing continent separation since thrust systems may act as major faunal barriers within a single tectonic plate. Other factors to consider when discussing plate separation include paleoclimatic belts.

## Plain language summary

Previous paleontological studies have used the differences in faunas (in the present case, now extinct fossil occurrences of trilobites) between two or more continental blocks (presently the Svalbard Archipelago, North America, and Scandinavia) to infer separation of these blocks by large distances up to several thousands of kilometers ca. 550 to 420 million years ago due to global plate tectonics processes. The present study shows that this method is biased because previous studies undermined factors such as climatic belts and (topographic) faunal barriers such as large, mountain-building cracks in the Earth's crust. The study builds on previous work on seismic data in the Barents Sea and Svalbard, which identified continuous, thousands of kilometers long, tens of kilometers thick networks of cracks extending from northwestern Russia to Svalbard and potentially northern Greenland, which formed 650 to 550 million years ago, therefore demonstrating a connection between all these continental blocks at 550-420 Ma.

## Introduction

Paleontological constraints have been extensively used in trying to understand plate tectonics over the past 100 years, *e.g.*, von Ubisch ( 1921, 1928), Eckhardt (1922), Colosi (1925), and de Beaufort (1925) who were some of the first scientists to use paleontological records of South America and western Africa to support Wegener’s Continental Drift theory ( Wegener, 1929). It is now widely accepted that South America was juxtaposed to western Africa in the late Paleozoic–Mesozoic, forming part of the supercontinent named Pangea and, thus, explaining similar paleontological records in upper Paleozoic–Mesozoic sedimentary rocks on both continents ( *e.g.*, Cisneros *et al.*, 2015; Modesto, 2006; Trewick, 2017). Similarly, faunal analyses already by Lemoine (1911) showed that Madagascar remained relatively close to eastern Africa until the mid-Cenozoic, while India had already been rifted away.

**Figure 1.**
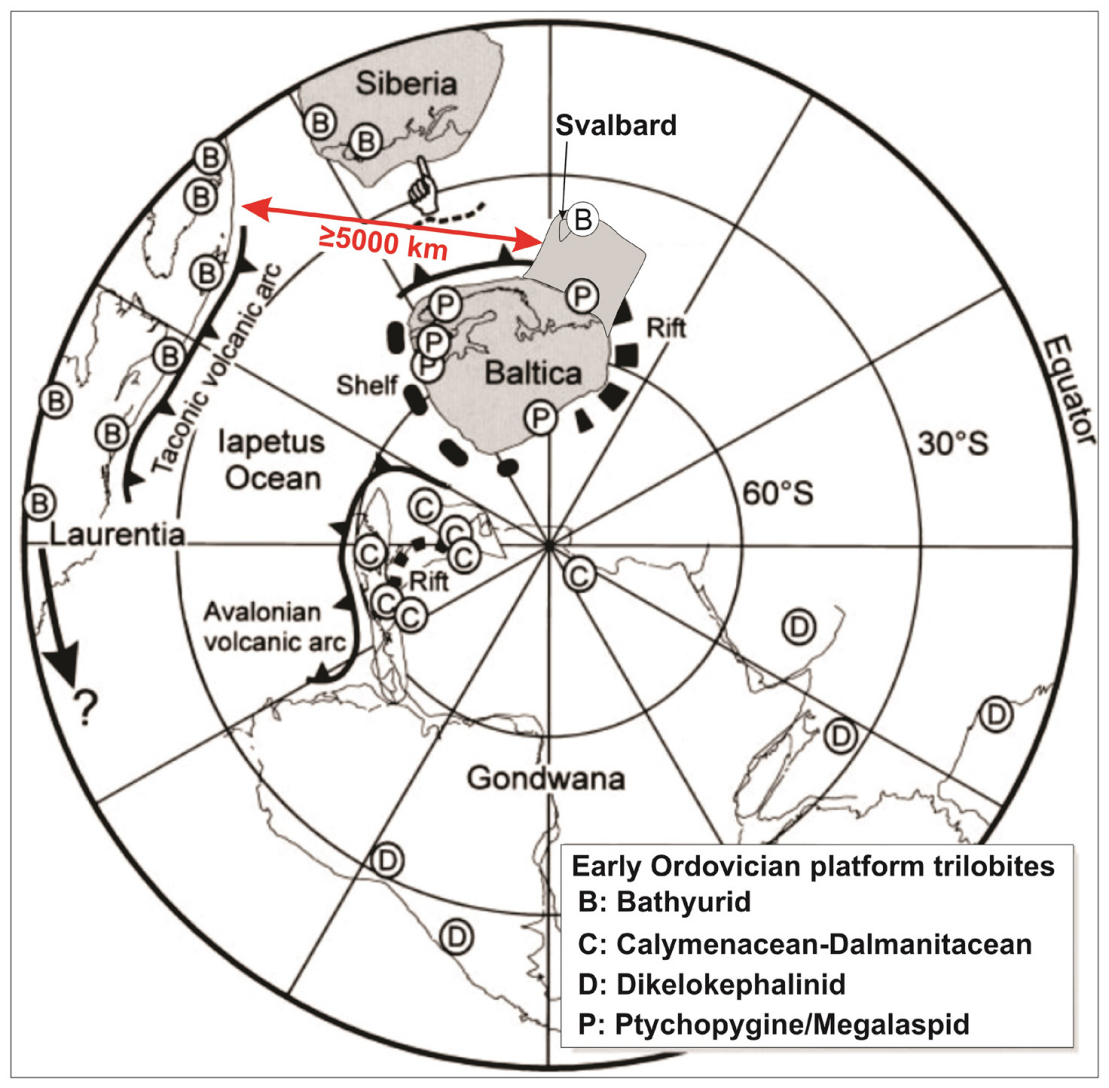


Later on, paleontological records were further used to infer land or sea connections between continents ( *e.g.*, Hansen & Holmer, 2011) and, even in some cases, estimate the minimum distance between two continents and the width of oceanic domains. This is the case of the Iapetus Ocean between Baltica and Laurentia, which was estimated to reach a maximum width of up to 5000 kilometers in the Ordovician, based on paleomagnetic and paleontological data ( Cocks & Torsvik, 2002; Domeier, 2016; Torsvik & Trench, 1991; Figure 1).

Paleogeographic and trilobite assemblages for relevant continents in the Early Ordovician modified after Smethurst *et al.* (1998). Notice the latitudinal change in the trilobite assemblages in Gondwana (including Avalonia), which is dominated by Calymenacean-Dalmanitacean trilobite in polar regions and by Dikelokephalinid at lower latitudes. The Svalbard Archipelago and the Barents Sea were added to Baltica based on the work by Koehl *et al.* (2022a) and Koehl *et al.* (2023). Also notice the c. 5000 km width inferred by previous works for the Iapetus Ocean between Baltica and Laurentia.

This is yet to be supported by paleomagnetic data, which are sparse and of poor quality in Baltica and inexistent in Svalbard for that period (e.g., Torsvik & Rehnström, 2001). In addition, paleomagnetic data only yields information on the latitude position of continents and are worthless to resolve longitudinal movements, which have been dominating the most recent Wilson Cycle (E–W seafloor spreading in the Atlantic, Pacific, and central Indian oceans). The resolution of paleomagnetic data (550 km; Butler, 1992) and occasionally aberrant behavior ( Abrajevitch & van der Voo, 2010) are other obstacles to the use of such data for paleogeographic reconstructions.

The use of faunal assemblages to infer the paleogeographic position of continental blocks is generally restricted to shallow-marine ( *e.g.*, Ordovician trilobites in Svalbard, Baltica and Laurentia; Fortey, 1984; Fortey & Bruton, 2013; Fortey & Cocks, 2003) or terrestrial groups ( *e.g.*, *Mesosaurus*; Modesto, 2006) since deep-marine faunas may spread over entire oceans ( *e.g.*, conodonts; Bergström, 1983; Wright & Stigall, 2013). Terrestrial and shallow-marine faunas are more prone to allopatric speciation by vicariance, *i.e.*, the isolation of a population by (a) geographic barrier(s) such as mountain ranges ( Trewick, 2017; Wright & Stigall, 2013). Such barriers are known to have broadly affected faunas in Laurentia in the Ordovician ( *e.g.*, onset of Taconian Orogeny; Wright & Stigall, 2013). However, recent studies show that vicariance events may also affect marine faunas for tens of millions of years. For example, in the past 25 Myr, the configuration of the continents formed major barriers (Terminal Tethyan Event, Isthmus of Panama, East Pacific Barrier), which prevented and in places still prevent the exchange of tropical faunas between the main biogeographical regions ( Cowman & Bellwood, 2013).

A key feature is the use made of Lower Cambrian and Lower Ordovician shallow water trilobite assemblages in Laurentia, Svalbard, and Baltica to infer terrane amalgamation and separation through time. In the early Cambrian, while Laurentia ( Fritz, 1972; Poulsen, 1974) and southwestern Spitsbergen showed remains of Olenellus svalbardensis Kielan ( Birkenmajer, 1978; Birkenmajer & Orlowski, 1977; Kielan, 1960; Major & Winsnes, 1955), the Cambrian trilobite record of Baltica was dominated by specimen of the Holmia, Schmidtiellus, and Kjerulfia genera (Holmiidea family, olenellid trilobite; Ahlberg *et al.*, 1986), together with ptychopariid trilobite ( Ahlberg, 1980). Similarly in the Early Ordovician, Greenland and northeastern Spitsbergen were dominated by a bathyurid trilobite assemblage, Baltica showed primarily asaphid trilobites (Megistaspidinae and Ptychopygiinae), which were used to propose the presence of a broad oceanic domain, the Iapetus Ocean between Laurentia–northeastern Spitsbergen and Baltica in the Ordovician ( Cocks & Torsvik, 2002; Domeier, 2016; Fortey & Bruton, 1973; Fortey & Bruton, 2013; Fortey & Cocks, 2003; Kröger *et al.*, 2017; Torsvik & Cocks, 2005; Figure 1). This model implies the presence of a major NE–SW-trending suture zone in the Barents Sea between Norway and Svalbard, which was suggested by previous studies based on Ocean Bottom Seismometer data ( Aarseth *et al.*, 2017; Barrère *et al.*, 2011; Breivik *et al.*, 2002; Breivik *et al.*, 2003; Breivik *et al.*, 2005; Gee & Teben’kov, 2004; Gee *et al.*, 2008; Gernigon *et al.*, 2014; Gudlaugsson *et al.*, 1998; Knudsen *et al.*, 2019; Krysinski *et al.*, 2013; Shulgin *et al.*, 2020). However, these monodisciplinary studies only considered the composition of the crust, and concrete evidence of such a major suture such as a fossil subduction zone and related structures ( *e.g.*, fold and thrust systems) is lacking. In addition, other fossil assemblages, though they generally agree to a proximity of all Svalbard’s basement terranes and North America, also show a similarity of fossil assemblages between Baltica and Laurentia in the Cambrian ( Ahlberg *et al.*, 1986; Babcock, 1994; Palmer & Peel, 1979; Poulsen, 1974) and between Baltica and northeastern Svalbard in the Ordovician ( Hansen & Holmer, 2011).

**Figure 2.**
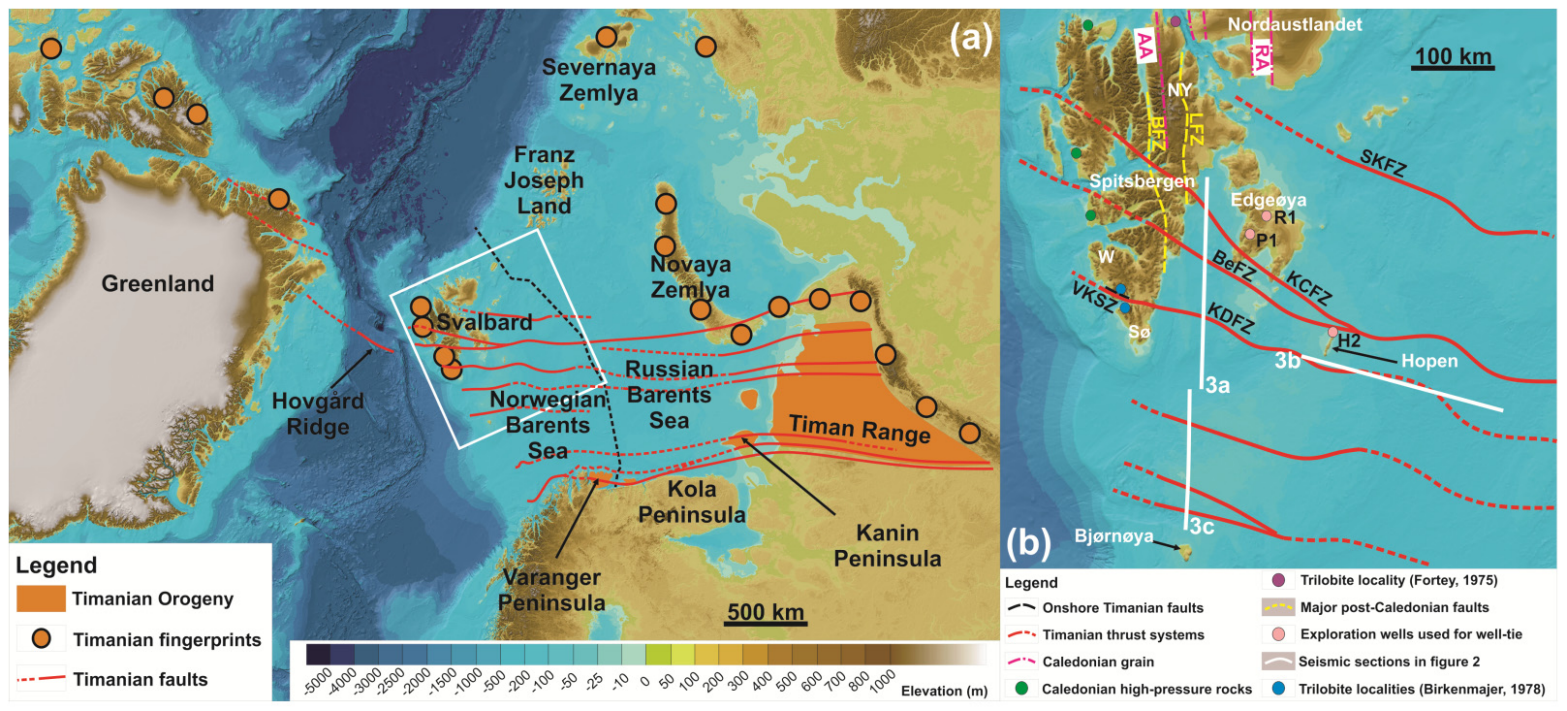
( **a**) Overview of Timanian thrust systems and fingerprints in the Norwegian Arctic showing the location of the study area (white frame). The dashed black line marks the boundary between the Russian and Norwegian Barents Sea. ( **b**) Overview of the study area in the Norwegian Barents Sea showing major Timanian thrust systems and the location of seismic profiles displayed in Figure 3. Timanian thrusts are from Koehl *et al.* (2022a), Koehl *et al.* (2023), Koehl (2024), and Koehl & Stokmo (2024). The basemap is the International Bathymetric Chart of the Arctic Ocean from Jakobsson *et al.* (2012). Abbreviations: AA: Atomfjella Antiform; BeFZ: Bellsundbanken fault zone; BFZ: Billefjorden Fault Zone; H2: Hopen-2 exploration well; KCFZ: Kongsfjorden–Cowanodden fault zone; KDFZ: Kinnhøgda–Daudbjørnpynten fault zone; LFZ: Lomfjorden Fault Zone; NY: Ny Friesland; P1: Plurdalen-1 exploration well; RA: Rijpdalen Anticline; R1: Raddedalen-1 exploration well; SKFZ: Steiløya–Krylen fault zone; Sø: Sørkapp Land; VKSZ: Vimsodden–Kosibapasset Shear Zone; W: Wedel Jarlsberg Land.

Recent analysis of seismic, magnetic, and gravimetric data throughout the Norwegian Barents Sea and the Svalbard Archipelago revealed the presence of several kilometers thick, deep, crustal-scale, hundreds–thousands of kilometers long, WNW–ESE-striking thrust systems, which display comparable top-SSW kinematics to and merge with Timanian fold and fault systems in the Russian Barents Sea, and onshore Novaya Zemlya and northwestern Russia ( Koehl, 2020; Koehl *et al.*, 2022a; Koehl *et al.*, 2023; Figure 2a). Such orogenic systems are also found onshore–offshore northern Norway ( Koehl & Stokmo, 2024). These thrust systems suggest that all terranes of the Svalbard Archipelago and the Barents Sea were already accreted to northern Norway at ca. 550 Ma and preclude the occurrence of large-scale strike-slip movements along major N–S-striking fault zones such as the Billefjorden Fault Zone during the Paleozoic ( *e.g.*, Harland *et al.*, 1974; Harland *et al.*, 1992; Labrousse *et al.*, 2008) because these would truncate the late Neoproterozoic Timanian thrust systems. Furthermore, the presence of Timanian grain is thought to extend beyond the Svalbard margin into the Fram Strait ( *e.g.*, Hovgård Ridge; Koehl, 2020), and possibly onshore northern Greenland ( Estrada *et al.*, 2018a; Rosa *et al.*, 2016; Figure 2a).

**Figure 3.**
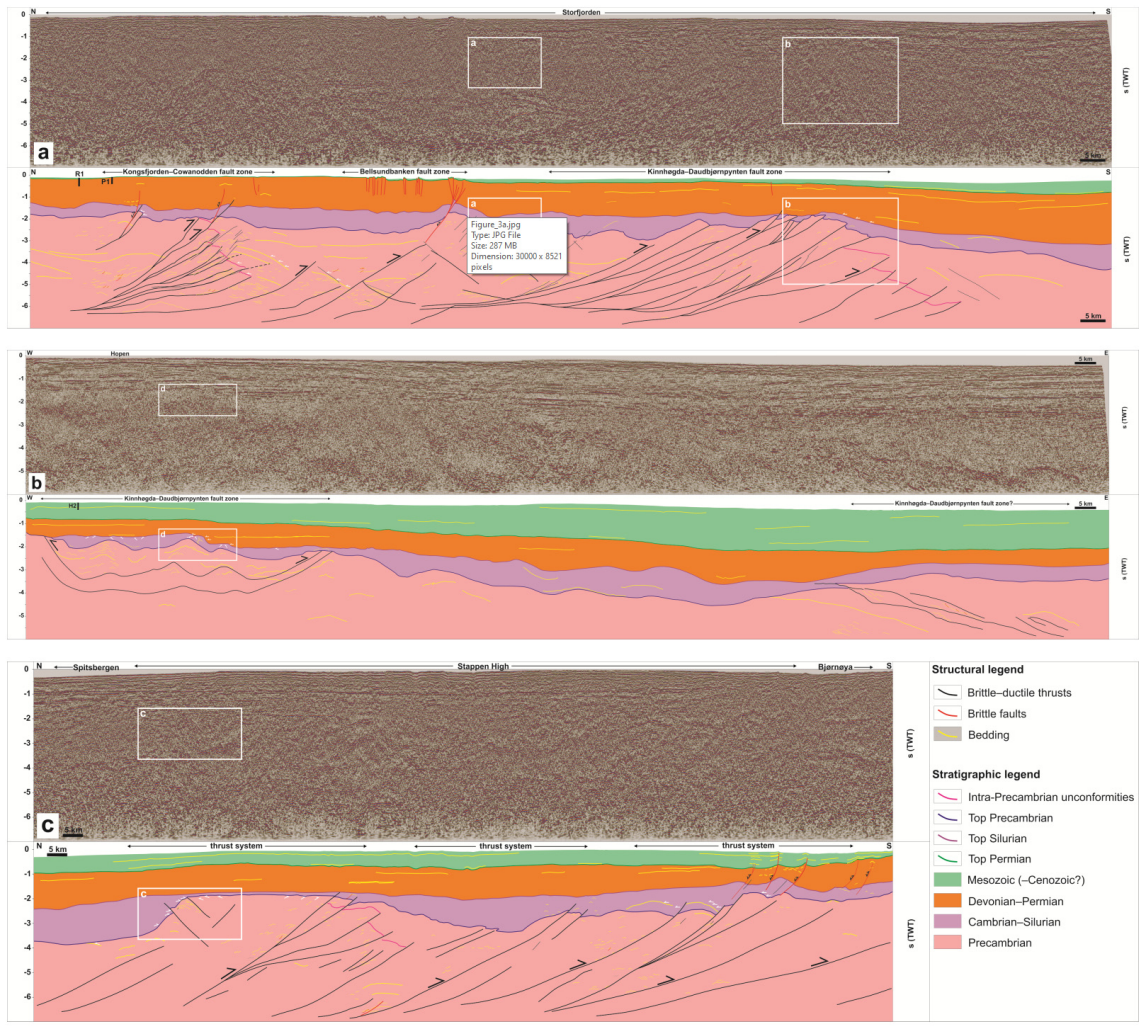
Seismic profiles ( **a**) in Storfjorden, ( **b**) south of Hopen, and ( **c**) between Bjørnøya and Spitsbergen. The profiles show several kilometers thick, crustal-scale, dominantly NNE-dipping Timanian thrust systems (black lines) within Proterozoic basement rocks, and related overprints within lower Paleozoic, upper Paleozoic, and Mesozoic (–Cenozoic?) successions. The profiles also show major (erosional) unconformities between the Proterozoic basement, lower Paleozoic, and upper Paleozoic successions (white half-arrows). The projected location of exploration wells to which the interpretation was tied was added to ( **a**) and ( **b**). The projection was made along a WNW–ESE- to NW–SE-trending axis for the Raddedalen-1 and Plurdalen-1 wells in ( **a**), i.e., parallel to the Kongsfjorden–Cowanodden fault zone, and along a NNE–SSW-trending axis for the Hopen-2 well in ( **b**), i.e., perpendicular to Timanian fault systems. Note that the wells were drilled onshore above sea level. The depth of the projected traces of the wells is therefore not the same as the original well. However, the thickness of sediments penetrated by the wells is accurate and was time-converted to be directly compared with the interpreted seismic profiles (see Koehl *et al.*, 2022a their supplement S3 for more details). The white rectangles indicate the location of Figure 4a–d.

**Figure 4.**
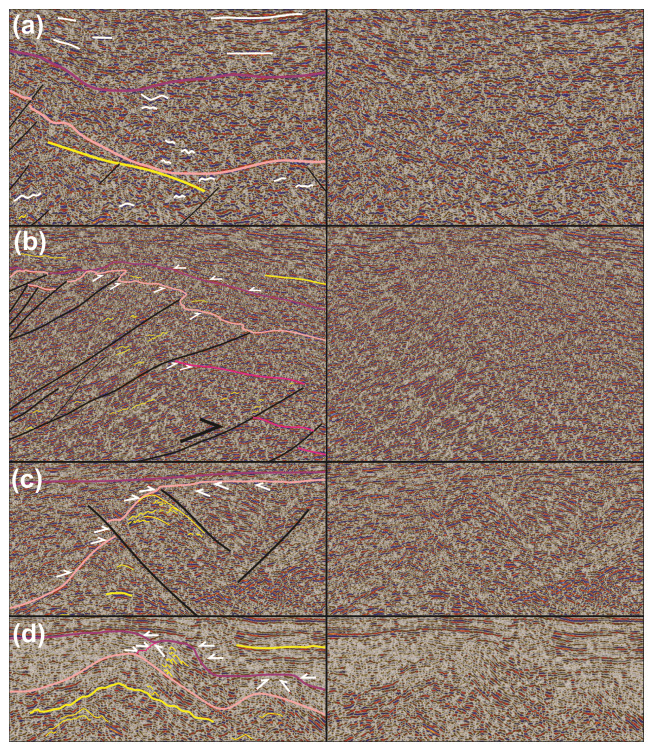
( **a**) Zoom in seismic data showing the undulating geometry of reflection characterizing Proterozoic basement and lower Paleozoic successions, whereas reflections within upper Paleozoic succession are relatively flat lying (white lines). ( **b**) Zoom in seismic data showing erosional truncation geometries in moderately NNE-dipping reflections below the fuchsia and pink reflections in the north, and the onlapping character of reflections at the base of the upper Paleozoic succession over the lower Paleozoic reflection (white half-arrows). ( **c**) Zoom in seismic data Between Bjørnøya and Sørkapp showing the onlapping character of reflections within the lower Paleozoic succession onto a Proterozoic basement paleo-high (white half-arrows) and early Paleozoic reactivation of an inherited Timanian thrust that offset the base of the lower Paleozoic succession in a reverse fashion. ( **d**) Zoom in seismic data showing erosional truncation geometries near the top of the lower Paleozoic succession and onlap geometries at the base of the upper Paleozoic succession (white half-arrows). See location of ( **a**– **d**) zooms in Figure 3. The legend is identical to Figure 3, except where specified otherwise. The location of ( **a**– **d**) is shown as white rectangles in Figure 3a–c.

The present contribution builds on the discovery of continuous Timanian thrusts throughout the Barents Sea and the Svalbard Archipelago by Koehl *et al.* (2022a) and discusses the importance of Timanian thrusts in these areas ( Figure 2b) on the use of paleontological records in plate tectonics reconstruction, especially when used to estimate the distance between two continents and determine terrane amalgamation and separation. The present contribution explores the late Neoproterozoic–early Paleozoic history of the Svalbard and the Barents Sea through analysis of the seismic reflection data and discusses the role of tectonic structures as potential major biogeographical boundaries. For information on the geophysical data (which includes both 2D and 3D seismic reflection data, gravimetric and magnetic anomaly maps, and exploration wellbores, including well tie), the reader is referred to Koehl *et al.* (2022a) and Koehl & Stokmo (2024).

## Geological setting

The lower Cambrian trilobite record of both southwestern Spitsbergen and Laurentia show comparable trilobite assemblages, including notably occurrences of Olenellus svalbardensis Kielan ( Birkenmajer, 1978; Birkenmajer & Orlowski, 1977; Fritz, 1972; Kielan, 1960; Major & Winsnes, 1955; Poulsen, 1974), which belong to the Bonnia-Olenellus Zone of the Pacific trilobite province ( Birkenmajer & Orlowski, 1977; Cowie, 1974). In Svalbard, such fossils occur in the in the Olenellusbreen Member of the Vardepiggen Formation and in the Flakfjellet Member of the Blåstertoppen Formation in Wedel Jarlsberg Land and Sørkapp Land ( Birkenmajer, 1978; Birkenmajer & Orlowski, 1977; Fritz, 1972; Kielan, 1960; Major & Winsnes, 1955; Poulsen, 1974). Trilobite assemblages are supposedly different in Baltica and include mostly olenellid trilobites of the Holmiidea family (Holmia, Schmidtiellus, and Kjerulfia genera; Ahlberg *et al.*, 1986) and ptychopariid trilobites ( Ahlberg, 1980), which typically define the Baltic trilobite province. It was therefore proposed that southwestern Spitsbergen was located close to Laurentia, but was separated from Baltica by large distances in the early Cambrian.

Similarly, based on trilobite fossil assemblage similarities, the northeastern terrane of Svalbard ( *i.e.*, Ny Friesland and Nordaustlandet; see Figure 2b for location) is believed to have been located at comparable latitude and possibly adjacent to northeastern Greenland in the Ordovician ( Cocks & Torsvik, 2002; Fortey, 1975; Fortey & Bruton, 2013; Fortey & Bruton, 1973; Fortey & Cocks, 2003; Kröger *et al.*, 2017; Smith & Rasmussen, 2008). Cocks and Torsvik (2002) and Fortey and Cocks (2003) further argue that the presence of bathyurid trilobites in both areas and their absence on Baltica, together with the presence of megistaspinid trilobites on Baltica and their absence in Laurentia–Svalbard suggest a broad separation of both continents in the Ordovician.

Moreover, the island of Bjørnøya in the Barents Sea shows Lower–Middle Ordovician sedimentary strata analogous to stratigraphic equivalents in northeastern Greenland ( Smith, 2000; Smith & Rasmussen, 2008). These overlie sedimentary rocks of presumed late Proterozoic age unconformably, thus suggesting a significant hiatus in the latest Neoproterozoic–earliest Ordovician, which is also comparable to the stratigraphic setting in northeastern Greenland ( Smith, 2000; Smith *et al.*, 2004). These similarities are thought to reflect the proximity of Bjørnøya with northeastern Greenland in the Ordovician and, thus, that Bjørnøya was part of Laurentia at that time.

The Svalbard Archipelago (excluding Bjørnøya) is commonly believed to be divided into three terranes consisting of Proterozoic–early Paleozoic metamorphic rocks, which recorded different tectonothermal events (e.g., Harland, 1969; Harland *et al.*, 1992; Harland *et al.*, 1993). Svalbard’s three terrane are commonly thought to have accreted during the early–mid Paleozoic Caledonian and Svalbardian orogenies through hundreds–thousands of kilometers long movements along major N–S-striking faults like the Billefjorden Fault Zone ( Harland *et al.*, 1974; Harland *et al.*, 1992; Labrousse *et al.*, 2008). Similarly, the Barents Sea is thought to correspond to a composite continental terrane assembled and accreted with Baltica and Svalbard during the Caledonian Orogeny. The Iapetus Ocean suture is commonly thought to crosscut the Barents Sea in a NE–SW fashion between Svalbard and northern Norway as suggested mostly from Ocean Bottom Seismometer data ( Aarseth *et al.*, 2017; Barrère *et al.*, 2011; Breivik *et al.*, 2002; Breivik *et al.*, 2003; Breivik *et al.*, 2005; Clark *et al.*, 2013; Gee *et al.*, 2008; Gee & Teben’kov, 2004; Gernigon *et al.*, 2014; Gudlaugsson *et al.*, 1998; Knudsen *et al.*, 2019; Krysinski *et al.*, 2013; Shulgin *et al.*, 2020). Although Ocean Bottom Seismometer data are reliable to discuss the composition of the crust and, therefore, to infer the possible presence of suture zones at depth ( *e.g.,* Aarseth *et al.*, 2017; Breivik *et al.,* 2002; Breivik *et al.,* 2003; Breivik *et al.,* 2005), they do not provide much information about existing structures (including subduction-related structures such as folds and thrusts) and are not as reliable as interdisciplinary studies ( *e.g.,* Klitzke *et al.,* 2019; Koehl *et al.*, 2022a). Notably, recent interdisciplinary works and reviews suggest that Svalbardian tectonism did not occur in Spitsbergen ( Koehl, 2021; Koehl *et al.*, 2022b), that Svalbard’s terranes and the Barents Sea were already amalgamated in the latest Neoproterozoic during the Timanian Orogeny at 650–550 Ma, and, thus, that the Iapetus suture is located in western Spitsbergen ( Figure 2b), *i.e.*, significantly west of the Billefjorden Fault Zone ( Koehl *et al.*, 2022a). Recent works also invalidated the occurrence of large-scale strike-slip movements along N–S-striking fault zone in Svalbard and the Barents Sea ( Koehl & Allaart, 2021; Koehl *et al.*, 2022a).

The structural and tectonic study by Koehl *et al.* (2022a) provided for the very first time evidence of continuous, late Neoproterozoic (i.e., 650–550 Myr old) thrust systems throughout the Barents Sea and Svalbard, thus pinning these areas together since 650 Ma. The present study focuses on the implications of these thrust systems for paleontology and paleogeography and notably on the relationship between trilobite assemblages distribution and plate tectonic separation.

## Methods

The present study is based on the interpretation of seismic reflection data in the northern Norwegian Barents Sea and Svalbard, which are all from the Norwegian National Data Repository for Petroleum Data (DISKOS database) of the Norwegian Petroleum Directorate. Seismic data were tied to exploration wells on Edgeøya (Raddedalen-1 and Plurdalen-1 wells; Bro & Shvarts, 1983; Harland & Kelly, 1997) and Hopen (Hopen-2 well; Anell *et al.*, 2014). See Koehl *et al.* ( 2022a, notably their method chapter) for detailed information on the well tie and for further discussion on the stratigraphy. Petrel (version 2021.3) was used to interpret the seismic reflection data, and CorelDraw (version 2017) was used to design the figures. Alternative open-source software are OpendTect and GIMP respectively.

The present study uses new knowledge in the seismic facies and structural character on seismic data of intensely deformed Proterozoic basement and lower Paleozoic metasedimentary rocks in the Barents Sea (see description of these successions in Koehl *et al.*, 2022a; Koehl *et al.*, 2023) and principles of seismic stratigraphy (e.g., erosional truncation, downlaps and onlaps; Mitchum *et al.*, 1977) to segregate them from overlying unmetamorphosed upper Paleozoic sedimentary successions. In order to be able to distinguish the various structures described in the present manuscript, high-resolution versions of the figures are found in *Underlying data* ( Koehl, 2023).

## Results

### Proterozoic basement rocks

Proterozoic basement rocks typically show moderate–high-amplitude seismic reflections either arranged into up to 3–4 seconds (TWT) thick packages of moderately NNE-dipping reflections (see black lines in Proterozoic succession in Figure 3a–c), or into packages of gently undulating, typically poorly continuous reflections (see thin yellow lines in Proterozoic succession in Figure 3a–c and white lines in Proterozoic succession in Figure 4a). Reflections of the former packages terminate abruptly upwards within the Proterozoic succession or against lower–upper Paleozoic successions with erosional truncation geometries (see white half-arrows marking truncation by fuchsia reflections within Proterozoic succession in Figure 3a–c and Figure 4b). Reflections of the latter packages are either undulating gently with a similar wavelength as reflections of overlying lower Paleozoic succession (see thin yellow lines in Proterozoic succession in Figure 3a–c and Figure 4a), or truncated upwards by lower–upper Paleozoic successions ( *e.g.*, white half arrows in Proterozoic succession in Figure 3c and Figure 4c). In places, the Proterozoic basement succession is characterized by moderate–high-amplitude, flat-lying reflections with relatively high continuity of up to 20–25 kilometers (see thick, flat-lying yellow lines in Proterozoic succession in the footwall of the Kongsfjorden–Cowanodden Fault Zone in Figure 3a). For a more detailed description of Proterozoic basement rocks and interpretation of thrust systems and related structures, the reader is referred to Koehl *et al.* (2022a).

### Lower paleozoic rocks

The lower Paleozoic succession in the northern Norwegian Barents Sea and Svalbard Archipelago is typically 0.5–1 second (TWT) thick but reaches a thickness of c. 1.5 s (TWT) in places ( *e.g.*, between Spitsbergen and Bjørnøya; Figure 3c). This succession consists of gently undulating, low–moderate-amplitude seismic reflections (see thin yellow lines within lower Paleozoic succession in Figure 3a–b and white lines within lower Paleozoic succession in Figure 4a). On E–W-trending seismic sections, some of these reflections are truncated upwards by flat-lying continuous reflections of the upper Paleozoic succession, thus resulting in erosional truncation geometries (see white half arrows in Figure 3b and Figure 4d). By contrast, erosional truncation geometries are sparse in this succession in N–S- to NNE–SSW-trending seismic sections. Instead, reflections within the lower Paleozoic succession appear to onlap Proterozoic basement rocks and, in places, they are laterally juxtaposed against or even partly overlain by Proterozoic basement rocks ( *e.g.*, between Bjørnøya and Spitsbergen and in Storfjorden; Figure 3a and c and Figure 4c). Onlap geometries are consistently accompanied by thinning of the lower Paleozoic succession over Proterozoic basement highs, *e.g.*, between Bjørnøya and Spitsbergen where the succession shows a thickness << 0.5 second (TWT; Figure 3c and Figure 4c), or near Sørkapp and south of Hopen where it is completely absent in places ( Figure 3a–b and Figure 4b and d).

### Upper Paleozoic–Mesozoic sedimentary rocks

Upper Paleozoic–Mesozoic successions in the Barents Sea and Svalbard are characterized by relatively continuous and flat-lying reflections displaying both high and low amplitudes ( Figure 3a–c and Figure 4a–b). The reflections either onlap Proterozoic–lower Paleozoic successions (white half arrows in upper Paleozoic succession in Figure 3a–b and Figure 4b and d), or parallel the Top lower Paleozoic reflection ( Figure 3a–c and Figure 4a and c). Typical thickness of the upper Paleozoic succession is 1–1.5 second (TWT). The Mesozoic succession was largely eroded around the Svalbard Archipelago ( Figure 3a) but it reaches a thickness > 2 seconds (TWT) towards the east and southeast ( Figure 3b).

## Discussion

### Caledonian reactivation of Timanian thrusts and Proterozoic–early Paleozoic structural highs

Local erosional truncation geometries displayed by reflections within the lower Paleozoic succession against upper Paleozoic strata are interpreted as erosional unconformities and suggest that, in places, lower Paleozoic rocks in the northern Barents Sea were deposited and eroded prior to the Devonian ( Figure 3b and Figure 4d). However, north of Bjørnøya, the lack of erosional truncation within the lower Paleozoic succession and the extremely thin character of this succession (much thinner than 0.5 second TWT) suggest that the area was likely a topographic high during most of the early Paleozoic ( *i.e.*, non-deposition or deposition of a condensed succession; Figure 3c and Figure 4c). This is supported by the geometry of NNE- and SSW-dipping thrusts bounding the Proterozoic basement high. These thrusts propagate into overlying and adjacent lower Paleozoic rocks and offset the Top Proterozoic basement reflection, thus suggesting basement uplift due to minor top-SSW and top-NNE reactivation of Timanian thrust systems in the early Paleozoic ( Figure 3c and Figure 4c). This episode of tectonism most likely reflects Caledonian reactivation–overprinting of Timanian thrust systems in this area, evidence of which are found throughout the Barents Sea and northern Norway ( Koehl *et al.*, 2023; Koehl & Stokmo, 2024).

Similarly, offshore near Sørkapp, the lower Paleozoic succession is thinning dramatically and is even completely absent above portions of the Kinnhøgda–Daudbjørnpynten Fault Zone and does not show erosional truncation ( Figure 3a–b and Figure 4b). In addition, basement-seated thrusts showing reverse offsets of the Top Proterozoic basement reflection transported slices of Proterozoic basement rocks onto lower Paleozoic rocks, thus indicating early Paleozoic thrusting and deposition of (part of) the lower Paleozoic succession into narrow foreland and piggy-back basins ( Figure 3a and c and Figure 4b–c).

Reactivation of Timanian thrusts as sinistral-reverse faults during Caledonian contraction is also known from onshore southwestern Spitsbergen ( Faehnrich *et al.*, 2020; Koehl *et al.*, 2022a; Mazur *et al.*, 2009). There, the Vimsodden–Kosibapasset Shear Zone segment of the Kinnhøgda–Daudbjørnpynten Fault Zone, which is associated to amphibolite facies metamorphism of Timanian age in nearby basement rocks ( Majka *et al.*, 2008; Majka *et al.*, 2012; Manecki *et al.*, 1998) and a major erosional unconformity between Tonian–lower Cryogenian and upper Cryogenian–Ediacaran rocks ( Bjørnerud, 1990; Bjørnerud *et al.*, 1991; Wala *et al.*, 2021), was completely overprinted by Caledonian deformation in the Middle Ordovician–Silurian (462 ± 11 Ma and 424 ± 6 Ma; Faehnrich *et al.*, 2020). Nevertheless, smaller nearby shear zones, which are less prone to reactivation and overprinting and strike parallel to the Vimsodden–Kosibapasset Shear Zone, may have preserved records of Timanian movement ( Faehnrich *et al.*, 2020, their sample 16-73A), thus potentially illustrating the reactivation history of Timanian thrusts in the Norwegian Arctic. Note that the large-scale (hundreds of kilometers) strike-slip movements initially suggested by early studies of the Vimsodden–Kosibapasset Shear Zone were invalidated by the geometry of Timanian thrusts in Storfjorden ( Koehl *et al.*, 2022a) and by the probable continuation of Timanian thrusts in the Fram Strait ( Koehl, 2020; Koehl, 2024), northern Greenland ( Estrada *et al.*, 2018a; Rosa *et al.*, 2016), and Arctic Canada ( Estrada *et al.*, 2018b).

Other erosional unconformities exist onshore Svalbard between upper Neoproterozoic (Ediacaran) and lower Paleozoic rock successions. The northernmost is the unconformity observed between the Ediacaran Dracoisen Formation and the lower Paleozoic Kapp Sparre Formation in western Nordaustlandet ( Stouge *et al.*, 2011). Koehl *et al.* ( 2022a, their supplement S2b) identified the presence of a major Timanian thrust in adjacent portion of the northern Barents Sea, the Steiløya–Krylen fault zone. It is probable that this major fault was reactivated during Caledonian contraction in the early Paleozoic, thus explaining the occurrence of the unconformity in western Nordaustlandet.

Some interpreted late Neoproterozoic Timanian thrusts are overlain by wedges of lower Paleozoic rocks thickening towards the faults (e.g., Figure 3a and c and Figure 4b). These geometries potentially suggest syn-tectonic sedimentation along normal faults in the early Paleozoic. Some of these potential normal faults are associated with upwards-convex reflections geometrically similar to rollover anticlines (e.g., southernmost thrust system in Figure 3c). However, most of the wedges of lower Paleozoic rocks can be explained by Caledonian reverse reactivation of nearby Timanian thrusts during mid-Paleozoic Caledonian contraction (e.g., Figure 3a and Figure 4b) with an interpretation as foreland/piggy-back basins. This is particularly well illustrated in Figure 4a and c. Since most WNW–ESE-striking Timanian thrusts were not suitably oriented to accommodate E–W-oriented Caledonian contraction, many were not reactivated during the Caledonian Orogeny.

In addition, convex-upwards bedding reflections are found both within Proterozoic and lower Paleozoic (metamorphosed) basement rocks, some of which are onlapped by lower Paleozoic sedimentary rocks ( Figure 3c). This would imply continuous normal faulting during the late Neoproterozoic–early Paleozoic. This contrasts markedly with the occurrence of major thrust-cored basement highs in the area (e.g., northern thrust system in Figure 3c), contractional indicators within all basement-seated thrust systems in the study area (e.g., asymmetric folds, contractional duplexes, antiformal thrust stacks, and minor thrusts; Figure 3a–c and Koehl *et al.*, 2022a their Figure 4a–e), and reverse offsets of the lower Paleozoic rock succession in nearby areas ( Figure 3c and Figure 4c) together with onshore evidence for two major contractional events during this period (Timanian and Caledonian orogenies; Braathen *et al.*, 1999; Majka *et al.*, 2008; Mazur *et al.*, 2009).

Furthermore, some of the apparent traces of normal faulting occur within major Proterozoic basement highs, which were therefore deeply eroded in the early Paleozoic. Since Timanian mylonitic thrust systems represent major rheological discontinuities, it is highly probable that they contributed to shaping the relief carved by erosion in the early Paleozoic and created some depression, which were later passively filled with lower Paleozoic sediments.

### Use of trilobite assemblages to infer disconnection of Baltica and Svalbard in the Cambrian–Ordovician

The marked differences in trilobite assemblages in southwestern Spitsbergen and Laurentia (presence of Olenellus svalbardensis Kielan; Birkenmajer, 1978; Birkenmajer & Orlowski, 1977; Fritz, 1972; Kielan, 1960; Poulsen, 1974), and in Baltica (dominance of Holmia, Schmidtiellus, and Kjerulfia genera of the Holmiidea family; Ahlberg, 1980; Ahlberg *et al.*, 1986) led previous workers to suggest a proximity of the southwestern terrane of Spitsbergen with Laurentia and a separation by large distances with Baltica ( *e.g.*, Torsvik & Cocks, 2016). The lower Cambrian trilobites in Svalbard were found in shallow sea sediments (dolomite) of the Blåstertoppen Formation ( Birkenmajer, 1978).

In addition, bathyurid and megistaspinid trilobites found exclusively on Laurentia–northeastern Spitsbergen and Baltica respectively were used to suggest that these continents were disconnected in the Early Ordovician. Both groups are thought to have evolved mostly in shallow seas and to reflect shallow marine environments ( Fortey & Cocks, 2003).

**Figure 5.**
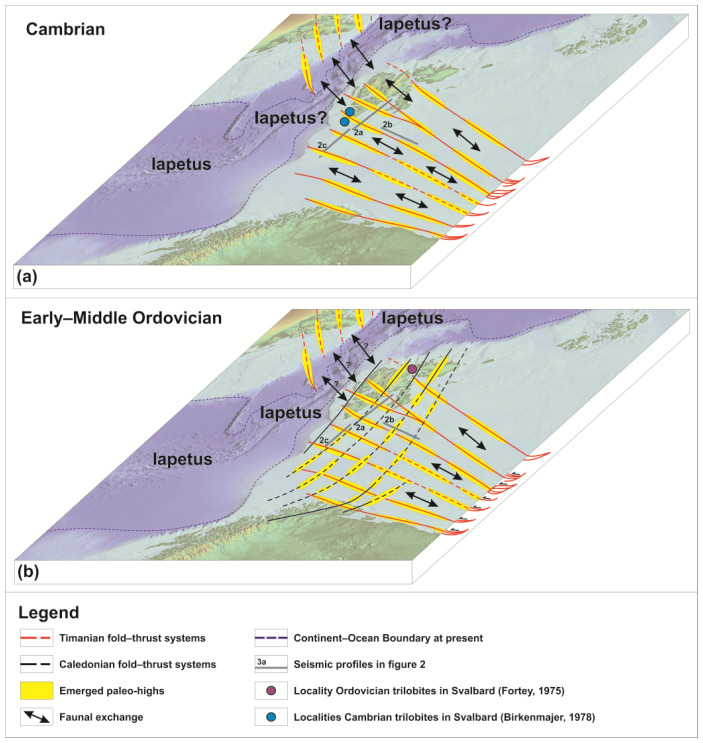
Conceptual model showing how emerged paleo-highs in the Barents Sea and Svalbard following ( **a**) preexisting Timanian thrusts in the Cambrian and ( **b**) both inherited (and reactivated) Timanian and newly formed Caledonian thrusts in the Ordovician controlled biological exchanges/mixing between Svalbard and Baltica in the early Paleozoic. Despite the opening of Iapetus, biological mixing between Greenland and Svalbard may have been possible until the Early Ordovician when top-east/southeast Caledonian thrusting and folding initiated, which was possibly compensated by transgression due to the closing of Iapetus ( Fortey, 1984). Present Continent–Ocean Boundary is from Dumais *et al.* (2020).

Seismic data in the northern Barents Sea and Svalbard clearly show that elongated, WNW–ESE-trending highs following reactivated–overprinted Timanian thrust systems existed in the early Paleozoic ( Figure 3a and c, and Figure 4b–c). Extremely thin to absent lower Paleozoic successions over these highs suggest that they were emerged above sea level for most of the early Paleozoic, *i.e.*, an environment not habitable by trilobites. These emerged WNW–ESE-trending highs formed in the late Neoproterozoic (–earliest Paleozoic?) and represented discrete topographical barriers between the southern (Baltica) and northern (Svalbard) portions of the continent. These barriers are thought to have prevented exchanges and mixing between trilobite communities of Baltica and Svalbard and to have acted as barriers between shelf faunas ( Figure 5a). This is further illustrated by the hiatus between uppermost Neoproterozoic and Early–Middle Ordovician sedimentary rocks onshore Bjørnøya ( Smith, 2000), which suggests that this island was largely emerged throughout the Cambrian and exposed to continental erosion, and by shallow marine fossil assemblages within Lower–Middle Ordovician rocks on the island indicating persisting shallow marine environment during the Ordovician. The presence of elongated highs in the Barents Sea is also supported by erosion or non-deposition of early–middle Cambrian deposits along NW–SE-trending highs in the Timanides of northwestern Russia ( Bogolepova & Gee, 2004).

Furthermore, the large number of WNW–ESE-striking Timanian thrusts in the Barents Sea suggest that, even if a few, N–S-trending, shallow marine connections ( *e.g.*, N–S-trending troughs) existed between the footwall and hanging wall of individual WNW–ESE-striking Timanian thrusts, dispersal of marine shelf faunas between Baltica and Svalbard–Laurentia would have been difficult due to the large number of topographical barriers ( *i.e.*, Timanian thrusts) between Baltica and Svalbard ( Figure 5a). Note that such barriers did not impede exchanges between northern Norway and northwestern Russia as suggested by comparable continental to shallow marine faunal assemblages in the Ediacaran–Cambrian ( *e.g.*, Desiatkin *et al.*, 2021; Högström *et al.*, 2013; Jensen *et al.*, 2018; Kolesnikov, 2019; Kolesnikov & Desiatkin, 2022). Additional obstacles to faunal mixing between Svalbard and Baltica may have been related to (1) climatic and environmental barriers due to the latitude difference between Svalbard, which was located at relatively low latitude comparable to Laurentia and Siberia (both of which also display bathyurid trilobite assemblage), and Baltica, which was located at mid to high southerly latitudes ( *e.g.*, Cocks & Torsvik, 2002; Cocks & Torsvik, 2021; Fortey & Cocks, 2003; Figure 1), and (2) to the onset of Caledonian folding and thrusting in the Early Ordovician in Baltica ( Eide & Lardeaux, 2002; Roberts *et al.*, 2002) and in Svalbard ( Dallmeyer *et al.*, 1990; Horsfield, 1972), hence further compartmentalizing the Barents Sea and preventing faunal exchanges between Baltica and Svalbard ( Figure 5b). Caledonian deformation both formed new N–S- to NNE–SSW-striking thrusts (e.g., Braathen *et al.*, 1999; Ohta, 1979; Ohta *et al.*, 1986; Witt-Nilsson *et al.*, 1998) and reactivated WNW–ESE-striking Timanian thrusts with minor dominantly sinistral strike-slip to reverse movements (e.g., Koehl *et al.*, 2022a; Koehl *et al.*, 2023; Mazur *et al.*, 2009), reworking some of them into NNE–SSW-striking folds and thrusts ( Koehl *et al.*, 2022a; Koehl *et al.*, 2023; Koehl & Stokmo, 2024). It is worth noting that transgressive events related to the closing of Iapetus may have partly compensated Caledonian folding and thrusting in the Ordovician in the north, therefore probably further allowing continuous exchange between Svalbard and Greenland ( Fortey, 1984).

A critical example of the described climatic and environmental barrier is illustrated by the distribution of Early Ordovician trilobite in Gondwana ( Smethurst *et al.*, 1998; Figure 1). Northern Africa and Avalonia were located near the South Pole and show Calymenacean–Dalmanitacean assemblages, whereas other parts of Gondwana located at lower latitudes (e.g., South America, Arabia, India, and Australia) show Dikelokephalinid assemblages ( Figure 1).

Fossil assemblages are still very useful in inferring connections between continents, *e.g.*, juxtaposition of South America and western Africa in the late Paleozoic–Mesozoic ( Cisneros *et al.*, 2015; Colosi, 1925; de Beaufort, 1925; Eckhardt, 1922; Modesto, 2006; Trewick, 2017; von Ubisch, 1921; von Ubisch, 1928) or a connection of northern Norway with northwestern Russia in the Ediacaran–Cambrian ( Desiatkin *et al.*, 2021; Högström *et al.*, 2013; Jensen *et al.*, 2018; Kolesnikov, 2019; Kolesnikov & Desiatkin, 2022), but the present study shows that the use of paleontological markers to infer disconnection between continents should be considered with care. In the present case, the Svalbard Archipelago was accreted to Baltica and to Laurentia in the latest Neoproterozoic during the Timanian Orogeny ( Koehl, 2020; Koehl *et al.*, 2022a). Svalbard remained attached to Baltica throughout the Paleozoic–early Cenozoic. In the early Paleozoic, Svalbard was separated from Laurentia by the Iapetus Ocean and later collided with Laurentia as suggested by blueschist and eclogite facies metamorphism of Caledonian age in western Spitsbergen ( Dallmeyer *et al.*, 1990; Horsfield, 1972; Kosminska *et al.*, 2014; Ohta *et al.*, 1995). However, the maximum distance between Svalbard and Laurentia at that time remains speculative. The Iapetus Ocean between Svalbard and Laurentia may have reached a width of several thousands of kilometers just like between Laurentia and Baltica ( *e.g.*, Domeier, 2016; Torsvik & Trench, 1991) or may have been significantly narrower. The fossil records on both continents simply suggest that exchanges of shelf faunas were possible between Svalbard and Laurentia in the early Cambrian–earliest Ordovician and, thus, that these two continents were possibly located close to each other and/or that they remained at a similar latitude (e.g., Figure 1 and Figure 5a–b). Blueschist–eclogite facies metamorphism in western Spitsbergen indicates that oceanic crust was subducted between Svalbard and Laurentia in the early Paleozoic, *i.e.*, that the suture of the Iapetus Ocean is most likely located in western Spitsbergen and that Svalbard and Baltica remained attached to each other throughout the Paleozoic, which is further supported by the identification of Timanian thrusts in the Loppa High and the southwestern Barents Sea ( Koehl *et al.*, 2023).

It is worth noting that other biotic assemblages such as acritarchs and chitinozoans do not yield the same results as trilobites when considering a disconnection between Baltica and Laurentia in the Cambrian–Ordovician ( Servais *et al.*, 2005; Slater *et al.*, 2017). Fortey and Mellish (1992) previously used biased arguments (e.g., unrevised dataset of acritarch species) to discredit the use of these groups ( Servais *et al.*, 2023). However, acritarchs and chitinozoans are now known to be just as valuable paleogeographic indicators as trilobites and show similar assemblages on both Baltica and Laurentia, thus suggesting a proximity of the two paleocontinents ( Servais *et al.*, 2005; Servais *et al.*, 2023; Slater *et al.*, 2017), *i.e.*, contrasting with the results from trilobite faunas.

The trilobite fossil record of Laurentia, Baltica, and Svalbard is also not without ambiguities. For instance, Poulsen (1974) and Palmer and Peel (1979) showed that specimens of the Holmia genera, which are representative of the Baltican trilobite province ( Ahlberg *et al.*, 1986), are also found in northeastern Greenland ( *i.e.*, Laurentia), therefore suggesting a link between Baltica and Laurentia in the early Cambrian rather than a separation by large distances.

Another potential bias is the lack of consideration of paleothermoclines, which appear to have controlled the distribution of trilobites in northern Greenland in the middle Cambrian ( Babcock, 1994). There, deep-water polymeroid trilobites show affinities to shallow, cool-water assemblages in paleocontinents located at high paleolatitude at that time, i.e., Baltica ( Babcock, 1994). The resemblance of northern Greenland deep-water fauna with Baltican fauna was initially used to ascribe an allochthonous character to middle Cambrian rocks in northern Greenland (i.e., exotic terrane). However, cool-water species were proven to be ubiquitous regardless of depth and latitude. In addtion, mixing of the Baltican and Laurentian trilobite assemblages occurred through gravity flow in northern Greenland, therefore invalidating the possibility of large tectonic transport of the rocks containing the Baltican assemblages in the middle Cambrian ( Babcock, 1994). These rocks were simply deposited at discrete levels in the water column (i.e., different temperature conditions and depositional environment). Babcock (1994)’s study not only suggests a strong influence of temperature (paleothermocline) on the distribution of fauna, but also an affinity of Laurentia and Baltica in the middle Cambrian, thus further questioning the use of trilobite assemblages to infer tectonic plate separation.

### Implications for plate reconstructions worldwide

The present study suggests that paleontological evidence, alone, is not a robust enough argument to infer long-distance separation of two continents or terranes. Consequently, many plate tectonics reconstructions, including recent ones, using the paleontological record as a discriminating factor should be reexamined. For example, Popov and Cocks (2017) using the faunal recruitment principle of Fortey and Cocks (2003) proposed a separation of all the Kazakh terranes by at least 1000 km from one another, and a similar separation (of the Kazakh terranes) with Siberia and Baltica in the early Paleozoic based on faunal assemblages, thus suggesting that the Kazakh terranes formed an archipelago several thousands of kilometers wide. Such enormous size is unrealistic as shown by the space problem it generates on plate reconstructions with other major continents located at similar latitude such as Baltica and Laurentia ( Domeier, 2018). It is therefore paramount to distinguish stand-alone discriminating factors and factors to be used in combination with others, and to establish clear guidelines as to what factors or combination of factors do warrant major continent/terrane separation.

The Earth’s sedimentary record represents only local and partial records of past faunal assemblages during specific time periods because of the non-deposition of sediments and their erosion in emerged areas for example. Let us image a distant future in which the fossil record of polar bears in Norway, Sweden and Finland was non-existent, due to for example non-preservation of polar bear remains in emerged areas of Norway and/or erosion of most if not all of the sedimentary record of the past few million years. This is reasonable because Norway does not show any onshore sedimentary record of the Cretaceous–early Cenozoic period for example ( *i.e.*, more than 100 Myr), whereas both Greenland and Svalbard show Cretaceous and early Cenozoic sedimentary strata ( *e.g.*, Dallmann, 2015; Stemmerik *et al.*, 1998; Svennevig, 2018). Let us also imagine that the sedimentary record from present-day and onwards in both Greenland, Norway, and Svalbard was preserved and captured the current distribution of polar bears in Arctic areas ( *i.e.*, in Greenland and Svalbard; Dupouy-Camet *et al.*, 2017). Following the faunal recruitment principle proposed by Fortey and Cocks (2003), paleontologists examining the fossil record of the present-day period and onwards in Greenland, Svalbard, and Norway millions of years from now could infer that the former two were likely part of the same tectonic plate and were disconnected from (not on the same plate as) the latter based on the presence of polar bear remains both in Greenland and Svalbard but not in Norway (see current distribution of polar bears in Dupouy-Camet *et al.*, 2017). This is erroneous because Svalbard belongs to the same tectonic plate as Baltica (Eurasian Plate), whereas Greenland belongs to the North American Plate. The faunal recruitment principle of Fortey and Cocks (2003) simply does not take into account environmental factors such as paleoclimatic belts, paleothermoclines, and major tectonic structures, which may play a significant role in the distribution of species and resulting fossil record.

## Conclusions

In the early Paleozoic, inherited Timanian thrust systems defined WNW–ESE-trending paleo-highs exposed to continental erosion in the northern Barents Sea. These highs acted as dispersal barriers for shallow marine faunas ( *e.g.*, Cambrian–Ordovician trilobites) that have been commonly used to infer continent–terrane separation in plate tectonics reconstructions. While the trilobite record suggests that Svalbard and Baltica were disconnected in the Cambrian–Ordovician, the presence of continuous, crustal-scale Timanian thrust systems throughout the Barents Sea and the Svalbard Archipelago indicates that Svalbard was accreted to Baltica in the latest Neoproterozoic and that these two continents remained attached to each other throughout the Paleozoic. The present study therefore suggests that paleontological records alone are not robust enough proxies to infer continent and/or terrane disconnection since other factors ( *e.g.*, major thrust systems, latitude differences, paleoclimatic belts, and paleothermocline) may play a significant role in preventing exchange and mixing between biological assemblages of aggregated continental plates during extended periods of time.

## Data Availability

The Two-Way Time (TWT) seismic reflection data used in the present study is under license by the DISKOS (Norwegian National Data Repository for Petroleum Data) database of the Norwegian Petroleum Directorate. The data may be accessed for research purposes and access can be requested by contacting the Norwegian Petroleum Directorate at https://www.npd.no/fakta/om-oss/kontakt-oss/. DataversNO: Replication data for: Implications of Timanian thrusts systems in the Barents Sea and Svalbard on using palontological constraints for plate tectonics reconstructions. doi.org/10.18710/BWZHL8 ( Koehl, 2023). This project contains the following underlying data: 00_ReadMe.txt. (detailed instructions to reuse the dataset and dataset relationship to existing contributions and datasets) Figure 1– Figure 5 (high resolution versions of the figures included in the present manuscript in jpg format. All copyright permissions granted).
