# Supplementary material for: Implications of Timanian thrust systems in the Barents Sea and Svalbard on using paleontological constraints for plate tectonics reconstructions
Source: Open Res Eur. 2024 Jul 12;3:189. Originally published 2023 Nov 7. [Version 2] doi: 10.12688/openreseurope.16674.2 (PMC11150901; doi:10.12688/openreseurope.16674.2)
Supplement: Peer review history

# Reviewer report: Reviewer response for version 2

**DOI:** 10.21956/openreseurope.19579.r42115

**Published:** 2024-07-30

**Authors:** Esther Izquierdo-Llavall

**Affiliations:** 1 Instituto Geológico y Minero de España, Zaragoza, Spain

I agree with the changes made by the author.

Is the study design appropriate and does the work have academic merit?

Yes

Is the work clearly and accurately presented and does it cite the current literature?

Yes

If applicable, is the statistical analysis and its interpretation appropriate?

Not applicable

Are all the source data underlying the results available to ensure full reproducibility?

Partly

Are the conclusions drawn adequately supported by the results?

Partly

Are sufficient details of methods and analysis provided to allow replication by others?

Yes

Reviewer Expertise:

NA

I confirm that I have read this submission and believe that I have an appropriate level of expertise to confirm that it is of an acceptable scientific standard.

# Reviewer report: Reviewer response for version 2

**DOI:** 10.21956/openreseurope.19579.r42114

**Published:** 2024-07-26

**Authors:** Alfonsa Milia

**Affiliations:** 1 National Research Council of Italy, Rome, Italy

The author has satisfied the requests. I ask to change the status of the manuscript to "approved"

 best wishes,

 Alfonsa Milia

Is the study design appropriate and does the work have academic merit?

Yes

Is the work clearly and accurately presented and does it cite the current literature?

No

If applicable, is the statistical analysis and its interpretation appropriate?

Yes

Are all the source data underlying the results available to ensure full reproducibility?

Yes

Are the conclusions drawn adequately supported by the results?

Yes

Are sufficient details of methods and analysis provided to allow replication by others?

Yes

Reviewer Expertise:

interpretation of seismic reflection profiles, stratigraphy and tectonics

I confirm that I have read this submission and believe that I have an appropriate level of expertise to confirm that it is of an acceptable scientific standard.

# Reviewer report: Reviewer response for version 1

**DOI:** 10.21956/openreseurope.18006.r40215

**Published:** 2024-06-04

**Authors:** Alexandre Kounov

**Affiliations:** 1 Basel University, Basel, Switzerland

This paper is presenting several seismic reflections profiles on the basis of which it was suggested the existence of a WMW-ESE trending thrust belt acting as a barrier for the shallow marine fauna between Svalbard Archipelago and Baltica from the Cambrian to the Ordovician. Such a scenario comes to refute the previously suggested idea of the existence of a large Iapetus ocean between these two crustal fragments.

 I think that the paper is generally well written and the presented data support well the suggested major conclusions. However, I have some remarks concerning the early Paleozoic tectonic evolution of the study area. It is not getting clear if the author suggests some active tectonics during the early Paleozoic responsible for the formation of the highs barrier or the relief is totally inherited from the Neoproterozoic Timanian thrusting.

 Details of my remarks and suggestions could be found in an annotated PDF file.:

Is the study design appropriate and does the work have academic merit?

Yes

Is the work clearly and accurately presented and does it cite the current literature?

Yes

If applicable, is the statistical analysis and its interpretation appropriate?

Yes

Are all the source data underlying the results available to ensure full reproducibility?

Yes

Are the conclusions drawn adequately supported by the results?

Yes

Are sufficient details of methods and analysis provided to allow replication by others?

Yes

Reviewer Expertise:

Structural geology, Tectonics

I confirm that I have read this submission and believe that I have an appropriate level of expertise to confirm that it is of an acceptable scientific standard.

# Response

**Published:** 2024-06-24

**Authors:** Jean-Baptiste Koehl

**Affiliations:** Geosciences, Universitetet i Oslo, Oslo, Oslo, Norway

Dear Dr. Kounov, thank you very much for your input on the manuscript, it is highly appreciated. Here is our reply to your comments. We hope the changes we implemented improve the shortcomings of the manuscript highlighted by your comments and suggestions. Please do not hesitate to contact us shall this not be the case for some comments. **Comments by the reviewer**

Comment 1: Plate tectonic reconstruction concept did not exist so far back in time.

Response: Agreed.

Changes: Replaced “plate tectonic reconstruction” by “trying to understand plate tectonics”.  

Comment 2: To much of “similar” words in this sentence.

Response: Agreed.

Changes: Replaced “similar” by “comparable”.  

Comment 3: Tell us a bit more about these terranes.

Response: Agreed.

Changes: Added “The Svalbard Archipelago (excluding Bjørnøya) is commonly believed to be divided into three terranes consisting of Proterozoic–early Paleozoic metamorphic rocks, which recorded different tectonothermal events (e.g., Harland, 1969; Harland *et al.*, 1992; Harland *et al.*, 1993).” and added Harland (1969) and Harland et al. (1993) to the reference list.  

Comment 4: Why within the Spitsbergen? Could you show on figure 1b where it could be? In figure 4 the Iapetus ocean is given between the Greenland and the archipelago.

Response: Agreed.

Changes: Added the location of high-pressure Caledonian rocks in western Spitsbergen and added reference to Figure 1b.  

Comment 5: The post-Timanian thrusts rather cut through the whole Lower Paleozoic sediments and I do not see evidence of syn-tectonic sedimentation. I would say from what I see  in the sections that the major Timanian thrusting continues in the early Paleozoic where the sedimentation was syn-tectonic with the formation of piggy-back basins. I see one big event of thrusting which is waning somewhere within the early Paleozoic. Unfortunately we do not have

Response: Disagreed. Thrusting mostly occurred in the late Neoproterozoic as documented by the minor propagation of Timanian thrusts into lower Paleozoic metasedimentary rocks (e.g., Figure 3b–c). Thus, only minor reactivation of Timanian thrusts occurred during the Caledonian Orogeny in the early–mid Paleozoic.

Changes: Replaced “top-SSW and top-NNE thrusting” by “minor top-SSW and top-NNE reactivation of Timanian thrust systems” and added “, evidence of which are found throughout the Barents Sea and northern Norway (Koehl *et al.*, 2023; Koehl and Stokmo, 2024” at the end of the paragraph.  

Comment 6: Here you confirm the early Paleozoic tectonics which is syn-sedimentational. This must be different from the Caledonian mid Paleozoic event.

Response: Disagreed. See response to comment 5.

Changes: See response to comment 5. Added “formed in the late Neoproterozoic (–earliest Paleozoic?) and” in the third sentence of the paragraph.  

Comment 7: Here you have the age of the major thrusting which led to the relief formation and erosion. An alternative scenario will be to have slow subsidence during this time span which is leading to the filing of a formed during the Neoproterozoic relief. By the early-middle Ordovician the whole relief was drowned and sediments started to be deposited everywhere. Would you believe in such scenario?

Response: Disagreed. The hiatus suggests exposure above sea level, which could be through either thrusting, or eustatic sea-level fall, or a combination of both during the latest Neoproterozoic because lower Paleozoic (meta) sedimentary rocks cap the Proterozoic basement highs (Figure 2 and Figure 3). Regional subsidence is possible to explain the flooding of the Proterozoic basement highs, but so is eustatic sea-level rise. Since this is not the focus of the present manuscript and does not impact the main conclusions, it is best to leave it out.

Changes: See response to comments 5 and 6.  

Comment 8: It seems to me too long and containing a lot of irrelevant information. I will suggest to shorten it to the most important message from the study area only.

Response: Disagreed. These are far-reaching implications of the present works, which could be applied to many other studies.

Changes: None.  

Comment 9: I am a bit puzzled. I am afraid I did not understand your idea. On page 7 in the Discussion you are talking about Early Paleozoic thrusting, basement uplift and deposition. From this I have understood that the highs were formed during this early Paleozoic tectonic event and therefore they are not inherited from the Timanian orogeny as you are stating here. Please make it clear in the whole text.

Response: Agreed. The thrust systems are Timanian and formed in the late Neoproterozoic. The early Paleozoic signal (reverse offset of early Paleozoic metasedimentary rocks; Figure 3b–c) represents a minor reactivation of the Timanian thrusts during Caledonian contraction. This was further specified in the text. See also response to comments 5 and 6.

Changes: See response to comments 5 and 6.

# Reviewer report: Reviewer response for version 1

**DOI:** 10.21956/openreseurope.18006.r38100

**Published:** 2024-05-28

**Authors:** Esther Izquierdo-Llavall

**Affiliations:** 1 Instituto Geológico y Minero de España, Zaragoza, Spain

This manuscript presents the interpretation of three seismic reflection profiles located in the eastern offshore of Svalbard. These profiles are in double time and two of them are N-S-striking whereas the third one is E-W striking; they do not intersect to each other. The interpreted seismic units and seismic facies are well-tied although none of the available exploration wells are located along seismic traces (from wells shown in figure 1). From the interpretation of seismic lines, authors propose a structural map of Timanian thrusts extending along the Russian and Norwegian Barents Sea. The map and the accompanying explanation highlights that these thrusts are along-strike continuous, with no major structural interruptions, which suggests there is no an ancient (post-thrusting) suture zone disrupting them (representing the suture of the Iapetus Ocean). At this point, previous paleontological data suggesting a ~5000 km separation between the Baltica and Svalbard (ancient Iapetus Ocean) during early Paleozoic times are discussed and the paleontological criteria used is questioned.

 The manuscript is well written and presented ideas are easy to follow. I personally found very interesting the way in which the author uses structural data to constrain plate tectonics models. The abstract and plain language summary are concise and clear. But the reader misses some relevant information at some points of the text/figures. Here there is a list of the main points that could be improved:

  

1. The presented seismic lines are located close to the eastern coast of Svalbard but the structural correlation of Timanian thrusts extends over hundreds/thousands of kilometers further to the East of this seismic information. It remains unclear how this structural correlation (which is key in the paleontological and tectonic plates model discussed) is done. If it is based on additional seismic/gravity/magnetic data from previous studies (as mentioned in the Introduction), it will be worth to show a map of magnetic/gravity anomalies and their trend or a map with the location of previously interpreted seismic profiles. Authors argue on the continuity of the thrust system, but readers would need to know how continuous (or discontinuous) the dataset used for inferring thrust geometries is.
2. A 5000 kilometers distance between Baltica and Laurentia in the Ordovician is suggested by paleontological and paleomagnetic data (said in the Introduction), but only paleontological data are questioned in the discussion. To complete this last part of the manuscript, it could be interesting to include some comments on the reliability of paleomagnetic constraints. Are available paleomagnetic data useful to infer the location of the Iapetus Ocean? Can they constraint the terrane paleolatitudes discussed in the manuscript?
3. Use of wells. Wells are not located over seismic traces but at a certain distance from them. Authors reference previous works to say that well tying was done, but it is probably useful for the reader to visualize (although projected) where these wells are on seismic profiles. Also, the reader misses some words on the depth of the wells. Are they reaching the pre-Cambrian and it is therefore a well-based knowledge of the seismic facies related to these basement units?
4. Interpretation of seismic profiles. Some of the thrusts affecting the Precambrian basement show a normal fault kinematics when the top Proterozoic horizon is examined. Besides, these apparently normal faults are consistent with thickness changes in overlying lower Paleozoic units (that thicken towards fault planes). This occurs in the southern part of figure 2c and central part of 2a. How are these thickness variations explained? It seems they indicate an extensional reactivation of basement faults during the lower Paleozoic which would have to be reconciled with the contractional reactivation described in relation to the basement high in figure 2c. Additionally, a word on the faults affecting upper Paleozoic units is missing. 
5. Figure 1: The horizontal scale and the reference on the structural mapping of Timanian thrusts are lacking. A label indicating the location of the island of Bjørnøya, which is mentioned in the text, can be included.
6. Figures 2-3. Indicate in the figure caption that inlets in figure 2 are shown in figure 3. Resolution of the seismic profiles (2 and 3) is low and makes difficult the evaluation of the quality of the seismic interpretation done. Even if the high-resolution profiles are published as underlying data, the author may try to play a bit with palette colors, brightness… to try to better capture the main features that are interpreted in the seismic profiles shown in the manuscript.   
7. Figure 4. From the text, the reader gets that WNW-ESE-striking Timanian thrusts were contractionally reactivated as WNW-ESE Caledonian thrusts. But figure 4 shows two perpendicular trends for Timanian-Caledonian structures?

I hope all these comments and suggestions will help the author to refine the present version of the manuscript.

Is the study design appropriate and does the work have academic merit?

Yes

Is the work clearly and accurately presented and does it cite the current literature?

Yes

If applicable, is the statistical analysis and its interpretation appropriate?

Not applicable

Are all the source data underlying the results available to ensure full reproducibility?

Partly

Are the conclusions drawn adequately supported by the results?

Partly

Are sufficient details of methods and analysis provided to allow replication by others?

Yes

Reviewer Expertise:

Structural geology and tectonics, paleomagnetism

I confirm that I have read this submission and believe that I have an appropriate level of expertise to confirm that it is of an acceptable scientific standard, however I have significant reservations, as outlined above.

# Response

**Published:** 2024-06-24

**Authors:** Jean-Baptiste Koehl

**Affiliations:** Geosciences, Universitetet i Oslo, Oslo, Oslo, Norway

Furthermore, some of the apparent traces of normal faulting occur within major Proterozoic basement highs, which were therefore deeply eroded in the early Paleozoic. Since Timanian mylonitic thrust systems represent major rheological discontinuities, it is highly probable that they contributed to shaping the relief carved by erosion in the early Paleozoic and created some depression, which were later passively filled with lower Paleozoic sediments.”

Comment 5: Figure 1: The horizontal scale and the reference on the structural mapping of Timanian thrusts are lacking. A label indicating the location of the island of Bjørnøya, which is mentioned in the text, can be included.

Response: Agreed. However, the location of the island of Bjørnøya is already displayed in Figure 1b.

Changes: Added “Timanian thrusts are from Koehl et al. (2022a), Koehl et al. (2023), Koehl (2024), and Koehl and Stokmo (2024).” in the caption of Figure 1 and a scale to Figure 1a and b.  

Comment 6: Figures 2-3. Indicate in the figure caption that inlets in figure 2 are shown in figure 3. Resolution of the seismic profiles (2 and 3) is low and makes difficult the evaluation of the quality of the seismic interpretation done. Even if the high-resolution profiles are published as underlying data, the author may try to play a bit with palette colors, brightness… to try to better capture the main features that are interpreted in the seismic profiles shown in the manuscript.

Response: Agreed. The author of the present manuscript has tried to manipulate the color palette and brightness to the figure’s advantage, without much success. The high-resolution versions of the figures are crucial to visualize the discussed features and are published on DataverseNO (Open Access data repository), as commonly done for studies of regional seismic reflection profiles by the author of the present manuscript (e.g., Koehl et al., 2022a; Koehl et al., 2023; Koehl, 2024; Koehl and Stokmo, 2024).

Changes: Added “The white rectangles indicate the location of Figure 3a–d.” in the caption of Figure 2, and “The location of ( a–d) is shown as white rectangles in Figure 2a–c.” in the caption of Figure 3.  

Comment 7: Figure 4. From the text, the reader gets that WNW-ESE-striking Timanian thrusts were contractionally reactivated as WNW-ESE Caledonian thrusts. But figure 4 shows two perpendicular trends for Timanian-Caledonian structures?

Response: This is correct. Some WNW–ESE-striking Timanian thrusts were reactivated during the Caledonian Orogeny and new N–S-striking thrusts formed during the Caledonian Orogeny and/or major Timanian thrusts were partly reworked into N–S- to NNE–SSW-striking fold-and-thrust systems (e.g., Koehl et al., 2022a; Koehl et al., 2023).

Changes: Added “(and reactivated)” in the caption of Figure 4 and “Caledonian deformation both formed new N–S- to NNE–SSW-striking thrusts (e.g., Ohta, 1979; Ohta *et al.*, 1986; Witt-Nilsson *et al.*, 1998; Braathen *et al.*, 1999) and reactivated WNW–ESE-striking Timanian thrusts with minor dominantly sinistral strike-slip to reverse movements (e.g., Mazur *et al.*, 2009; Koehl *et al.*, 2022a; Koehl *et al.*, 2023), reworking some of them into NNE–SSW-striking folds and thrusts (Koehl *et al.*, 2022a; Koehl *et al.*, 2023; Koehl & Stokmo, 2024).” to the discussion. Also added Ohta (1979), Ohta et al. (1986), and Witt-Nilsson et al. (1998) to the literature list.

# Reviewer report: Reviewer response for version 1

**DOI:** 10.21956/openreseurope.18006.r38105

**Published:** 2024-04-14

**Authors:** Alfonsa Milia

**Affiliations:** 1 National Research Council of Italy, Rome, Italy

The paper discusses how the presence of a thrust belt in the Barents Sea and Svalbard may have been a barrier for the spread of trilobites between different regions. The implications of this are fundamental for the palaeogeographic reconstruction of the area where, in contrast, the presence of an ocean as a barrier for the spread of trilobites had been assumed. The subject is interesting, and the “Smoking gun” is the documentation of emerged areas at that time. It is important to point out that: a) The story concerns the recognition of a thrust belt located in the area where the Iapetus ocean was hypothesized, during the pre-Paleozoic-Paleozoic period, in the Barents Sea-Svalbard. The hypothesis of an ocean in that area was based on the distribution of trilobites in the area and in particular on the differences in the Trilobite assemblage between Laurentia-Svalbard and Baltica. To understand the new proposed geological history, it is important to reconstruct the physiography of the area and of the emerged zones that may have functioned as a barrier for the distribution of the Trilobites. The broader outcome is the reevaluation of the role of paleontology in reconstructing the paleogeography of an area. b) The challenge is to individuate the erosional truncation, gaps in the succession that suggest the formation of the barriers at that time. c) The work is based on the interpretation of seismic reflection profiles.

 I suggest to eliminate: 1) the term “Sequence stratigraphy”, as this term is linked to the identification of systems tracts to be correlated with climatic fluctuations, and replace it with the term “Seismic stratigraphy”; 2) The term “Toplap” that is classically referred to the strata termination of the upper part of the progradational units; in this contest is more appropriate the use of “angular unconformity” or “erosional truncation”.

**Abstract:**

 Methods: I suggest to change in “The interpretation of seismic reflection profiles from the DISKOS database, calibrated with the boreholes drilled in the adjacent emerged areas, was used to reconstruct the geology of the area and in particular the buried thrust belt" The term "NEW INTERPRETATION" does not seem appropriate since the interpretation proposed by the author seems to be the same or very similar to the one published by the same author in 2022 (e.g. Koehl *et al.*, 2022 ^1^). However, a more detailed interpretation can be made in Figure 3, where some details of the seismic profiles can be seen (see the attached modified linked figure here, with an explanation of the annotations in the paragraph below):

 Regarding figure 3, the author should check the reference in the text relating to figure 3C and 3D which are perhaps reversed. Regarding the interpretation of the profiles, although the chain areas present complex structures that are difficult to interpret on seismic profiles, following the parallel sections of the reflectors, the angular discordances and the stratal terminations it is possible to propose more detailed interpretations ).

**Conclusion: **I suggest to change in “The individuation of emerged areas in correspondence of the thrust belt suggest that …. The results indicate that paleontological constraints….” This is because the subject of the paper is the analysis of the thrust belt using the interpretation of seismic profiles.

**Keywords:** insert “seismic reflection profiles” (the paper is based on the interpretation of seismic reflection profiles)

 The **Introduction **should focus on the palaeogeography of the area and the presence of an ocean in the palaeogeographical maps. Successively you can explain on what basis it was made, and introduce the discovery of the thrust belt, first assumed from the OBS data and then reconstructed using seismic profiles etc. Beginning with "Paleontological contraints have been ..." suggests the idea that this is paleontological work.

**Geological setting: **I suggest to add a figure with a paleogeography. I think this is fundamental for this work.

 The last sentence of the Geologic Setting “The present study focuses…” should be deleted.

**Methods: **Please add a figure with a stratigraphic column of the area or a well stratigraphy. What does “new knowledge in the seismic facies and structural character.” mean?

**Results:** I suggest reorganizing this paragraph by first describing the seismic units by identifying the 4 main units (e.g. A, B,C,D). Then, based on the geometric features and correlation with the stratigraphies of boreholes and outcrops, a geological attribution can be given (e.g. Age, lithology etc. Pre-Cambrian, Cambrian-Silurian, Devonian- Permian, Mesozoic).

 I recommend talking about thickness in seconds. However, if it is necessary to report in kilometers it would be helpful to indicate the speeds used for the transformation from seconds to kilometres.

**Discussion: **Please change in “CALEDONIAN REACTIVATION OF TIMANIAN THRUST AND EVIDENCE OF PHYSIOGRAPHIC BARRIERS”

 Please change “Local toplap geometries…….” in “Angular unconformities …………………suggest that………. where **deformed** and eroded prior to the Devonian”.

 The lack of an angular unconformity and a thin succession do not document a continental erosion! However, these characteristics in correspondence of a structural high due, for example, to the nappes superposition suggest the formation of a submarine or continental physiographic barrier.

**Figure 1:** Order caption: toponyms (NY, W), wells (P1, R1), fault zones/anticlines (AA, RA, BeFZ, BFZ, etc.).

Is the study design appropriate and does the work have academic merit?

Yes

Is the work clearly and accurately presented and does it cite the current literature?

No

If applicable, is the statistical analysis and its interpretation appropriate?

Yes

Are all the source data underlying the results available to ensure full reproducibility?

Yes

Are the conclusions drawn adequately supported by the results?

Yes

Are sufficient details of methods and analysis provided to allow replication by others?

Yes

Reviewer Expertise:

interpretation of seismic reflection profiles, stratigraphy and tectonics

I confirm that I have read this submission and believe that I have an appropriate level of expertise to confirm that it is of an acceptable scientific standard, however I have significant reservations, as outlined above.

## References

[1] : The timing of the Svalbardian Orogeny in Svalbard: a review. *Solid Earth* .2022;13(8) : 10.5194/se-13-1353-2022 1353-1370 10.5194/se-13-1353-2022
